# Sensor Fault-Tolerant Control Design for Magnetic Brake System

**DOI:** 10.3390/s20164598

**Published:** 2020-08-16

**Authors:** Krzysztof Patan, Maciej Patan, Kamil Klimkowicz

**Affiliations:** Institute of Control and Computation Engineering, University of Zielona Góra, 65-516 Zielona Góra, Poland; M.Patan@issi.uz.zgora.pl (M.P.); k.klimkowicz@issi.uz.zgora.pl (K.K.)

**Keywords:** braking control, nonlinear systems, fault tolerant control, fault detection, iterative learning control, neural networks

## Abstract

The purpose of the paper is to develop an efficient approach to fault-tolerant control for nonlinear systems of magnetic brakes. The challenging problems of accurate modeling, reliable fault detection and a control design able to compensate for potential sensor faults are addressed. The main idea here is to make use of the repetitive character of the control task and apply iterative learning control based on the observational data to accurately tune the system models for different states of the system. The proposed control scheme uses a learning controller built on a mixture of neural networks that estimate system responses for various operating points; it is then able to adapt to changing working conditions of the device. Then, using the tracking error norm as a sufficient statistic for detection of sensor fault, a simple thresholding technique is provided for verification of the hypothesis on abnormal sensor states. This also makes it possible to start the reconstruction of faulty sensor signals to properly compensate for the control of the system. The paper highlights the components of the complete iterative learning procedure including the system identification, fault detection and fault-tolerant control. Additionally, a series of experiments was conducted for the developed control strategy applied to a magnetic brake system to track the desired reference with the acceptable accuracy level, taking into account various fault scenarios.

## 1. Introduction

Modern industrial systems are usually complex and nonlinear. This leads directly to challenging problems related to control design as requirements imposed on the control quality and robustness continuously increase. Since it is not easy to deal with the nonlinear models, there is still a need for new systematic methods taking advantage of the specificity of a particular class of nonlinear systems.

A magnetic brake also called an eddy-current brake is a typical example of such nonlinear systems with tangible applications in various areas of the automotive industry, including high speed railways, big trucks and industrial elevators. Basically, in the regime of the high speed reached by current vehicles, the classical friction-based retarders become ineffective and insufficient. Therefore, magnetic or electro-magnetic brake control systems constitute an attractive alternative to ensure travelers and vehicle safety and reliability. These are characterized by the fast response time, a non-contact construction, and a relatively low level of failure rate in comparison to conventional brakes.

As for control design for magnetic brakes, the problem is considered to be difficult since the complex dynamics include not only temporal but also spatial effects. This excludes the direct extension of results from control theory for linear systems. Additionally, the system is characterized by a strict antisymmetry of control, as one can control only the rate of deceleration. Nevertheless, a few more or less successful attempts at adapting this theory have been reported by various authors in the control engineering literature.

In [1], the authors used an input–output feedback linearization scheme for speed control. However, they used a simplified control-affine model to derive the control law. In [2], the author used the classical proportional-integral (PI) controller with the current reference provided by means of the Kriging method. However, the optimization problem was solved using a genetic algorithm, which is a time-consuming method. In [3], the authors applied an adaptive control of vehicle speed. However, they assumed a low order lumped-parameter model of the plant. In [4], the authors applied a sliding mode control to design a speed control that is robust for changing road conditions, but again, they used a simplified approximated model as proposed in [1]. In turn, a torque tracking control by means of a sliding model was proposed in [5]. The author applied a simple static polynomial-based torque model, which leads directly to excessive sensitivity on the changes of the operating point of the control system during the implementation of this technique.

All reported papers used the simplified models of the magnetic brake and described closed-loop control schemes. The main problem that arises in the case of nonlinear control system design is derivation of the exact model of the plant considered. From a physical point of view, the magnetic brake is a distributed-parameter system, requiring sophisticated mathematical modeling that takes into account its spatiotemporal dynamics. This makes the accurate modeling numerically difficult and time-consuming, which constitutes one of crucial design difficulties.

In addition to this, there is a significant lack of contributions discussed in the context of fault-tolerant control. In fact, there are just a few papers covering this issue in the context of breaking control for electromagnetic systems. An adaptive fault-tolerant control based on a Takagi–Sugeno fuzzy model and observer design is reported in [6], but it consists of sequential recalibration of Kalman filters, reverting this approach to the linear approximation of the system. Another approach to achieve fault-tolerant brake control utilized the brake-by-wire strategy [7]. However, it was strongly dependent on preset recovery strategies. Additionally, it focused on the nonlinear effects of contact force control and did not take into account the specificity of the electromagnetic brakes, limiting its applicability in this area. In [8], the braking controller was constructed for vehicles with regenerative in-wheel electromagnetic brakes using sliding mode control and a fault-tolerant control architecture included as an adaptive scheme. However, in this case, the main stress was put on the vehicle dynamics and again the linear model of braking torque was applied. On the one hand, all of the approaches mentioned above allow to avoid significant perturbations in the control and provide satisfactory results in some particular situations; on the other hand, they do not take into account the effects of the complex dynamics of brakes, neglecting the nonlinearity that may introduce an additional level of uncertainty and significantly affect the reliability of fault detection. To fill this gap, this work focuses on an alternative control strategy driven by measurement data and developed on the basis of an iterative learning controller enhanced with statistical hypothesis testing for fault detection.

If an accurate mathematical model of the system is not available, the reasonable alternative is to design the model using input–output data recorded in the real plant during replications of the control task. In this context, iterative learning control (ILC) is a suitable technique to achieve ideal tracking of the given reference profile [9]. The learning controller calculates the current control signal based on the archive control data available from previous experiments. Most often, a learning controller has a fixed structure along the trial domain. However, in practice, a controller should be robust to disturbances and the faults within the infrastructure of the plant [10]. Therefore, a learning controller design should include the above circumstances into the analysis. In this paper, a neural network-based iterative learning control scheme is proposed, which is capable of adapting its own behavior to the changing working conditions of the plant (related to potential sensor faults) through a data-driven training process. In the background of ILC, the additional advantage is that the squared tracking error norm stands for the convenient statistic for the fault detection task. It makes it possible to use classical parametric hypothesis testing on the abnormal state of the system, leading to a simple yet efficient thresholding rule for sensor fault detection.

Due to technological developments, modern industrial plants are more and more complex and are composed of an ever-growing number of interacting devices, including a huge number of measuring devices. That is the reason that industrial plants are becoming very vulnerable to any deviation from the so-called normal operating conditions caused by unpermitted deviations of plant characteristics or unexpected changes of system variables. Such a deviation is called a fault. Early detection of faults can provide a way to avoid a shut-down or even a failure of the system. More importantly, a proper fault accommodation is strongly related to avoiding the large financial losses and serious injuries or even death of personnel. On this basis, fault-tolerant control (FTC) has come into prominence and has received increased attention in the last decade [11,12,13,14,15]. The FTC approaches can be be split into passive and active ones. Passive FTC uses a priori knowledge about anticipated faults that can affect the system. In turn, active FTC methods use the information acquired from a fault diagnosis subsystem in order to accommodate a fault. An important class of fault tolerant systems is sensor FTC. The simple and intuitive way is to introduce hardware redundancy, which, however, complicates the construction of the system and increases maintenance costs. A more flexible solution is to use the idea of a virtual sensor [16,17,18]. The idea is to exclude the measurements given by the faulty sensor and replace them with data provided by the virtual sensor. The virtual sensor requires a fairly accurate plant model. In this paper, we propose to apply a mixture of neural network models, which renders it possible to effectively accommodate a sensor fault. This strategy is called a fault hiding approach [19], which renders it possible to apply the same controller to both faulty and fault-free cases.

The contributions of the paper can be stated as follows.

1. Proposing a sensor active FTC system for magnetic brakes based on iterative learning control.
2. Developing a model of a magnetic brake by means of the mixture of state-space neural network models and gain scheduling.
3. Performing fault accommodation analysis for various types of sensor faults.

The paper is organized as follows. In Section 2 the magnetic brake system is described. In Section 3 the control scheme using iterative learning control is provided and the control performance for normal operating conditions is also discussed. Section 4 is devoted to the modeling issues. State-space neural networks are employed to model the plant at different operating points and the overall model of the plant is achieved by means of gain scheduling in the form of a mixture of models. Section 5 introduces the idea of sensor active FTC, including fault detection and accommodation and FTC in case of different sensor faults. The last section summarizes the paper.

## 2. Magnetic Brake

**Figure 1:**
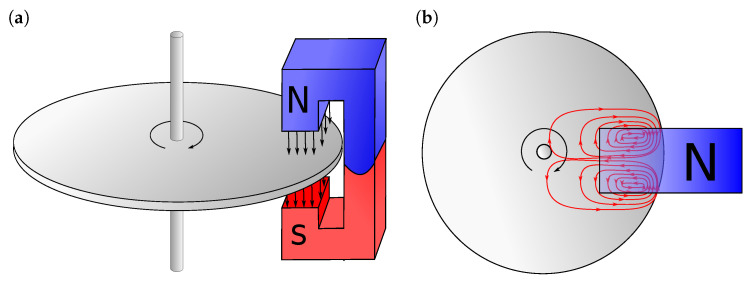
(**a**) Schematic view of a magnetic brake and (**b**) upper view with eddy currents field.

A magnet brake, in simple terms, consists of a disk of conductive material and a magnet generating a magnetic field in which the disk is rotating. The simplest form of the device is depicted in Figure 1 (left panel).

When a conductor moves in a magnetic field, it induces eddy currents, cf. Figure 1 (right panel), which interact with the magnetic flux to produce Lorentz forces resulting in a braking torque slowing down the disk. Let $D\in R^{3}$ denote a spatial domain representing the disk. Then, the evolution of the rotational velocity ω of the disk over the time interval $\left(0,t_{f}\right)$ can be described by the following initial value problem:

(1) $$J\frac{d\omega (t)}{dt}=M\left(B_{0}\left(t\right),\omega \left(t\right)\right),\;\;\omega \left(0\right)=\omega_{0},$$

where *J* is a disk moment of inertia and $B_{0}\left(t\right)$ is the magnetic flux of the external magnet (or electromagnet). *M* is a total torque affecting the disk:

(2) $$M=\int_{D}T_{q}\left(B\left(x,t\right),B_{0}\left(t\right),\omega \left(t\right)\right)\;dV,$$

where B(x,t) is the spatiotemporal field of internal magnetic flux inside the disk, dV is a volume element of the spatial domain and $T_{q}$ denotes a Lorentz force on the volume element related to the spatial location *x*.

It becomes clear that the eddy current brakes are nonlinear systems with responses depending on the spatiotemporal dynamics of changes in magnetic flux ([20], Chapter 9). It is strongly dependent on the shape of the domain D as well as the form of the external magnetic flux generated by the magnet. Hence, the modeling with typical methods for distributed-parameter systems, such as finite or boundary element methods, are not only challenging but also difficult to apply in practical control design, as any change to the spatial distribution of external magnetic fields (e.g., reallocating the magnets or changing the boundary conditions) requires a total rebuilding of the model.

In fact, the accurate estimation of the braking torque is one of the key challenging problems for both the design of control systems and fault detection and its compensation. Furthermore, the modeling uncertainty has to be taken into account, as it is an important factor in applications to real-world engineering systems. Fortunately, Equation (2) defining the torque integrated over the spatial domain of the disk can be approximated up to a satisfactory accuracy based on the measurement data. Here, we make use of the well-known ensemble averaging properties of neural networks. They not only provide an important alternative for accurate modeling of the nonlinear torque, but also deliver necessary robustness with respect to disturbances as they can generalize the system response. Additionally, in order to introduce these uncertainties into control synthesis, the iterative learning control is applied, being considered a well-known robust control design technique. This is especially valid in the efficient control design, which will be discussed in the following section.

For the purpose of the simulation study considered in this work, we used an aluminum disk with a radius of 10 cm and a thickness of d=1 mm. The disk is moving in the external magnetic field generated by an electromagnet with a maximum flux of 0.1 T. This represents a typical configuration encountered in analog energy meters.

**Figure 2:**
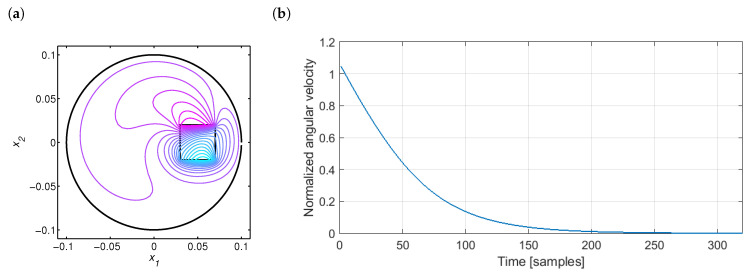
(**a**) Magnetic flux density *B* for ω=200 rpm and $B_{0}=0.1$ T, and (**b**) required reference profile.

The control objective is to produce the input signal of magnetic flux $B_{0}\left(t\right)$ leading to the desired profile of output angular velocity ω(t) with the initial value of 200 rpm. As the control algorithm, the iterative learning control portrayed in detail in Section 3 was used. The exemplary spatial distribution of the magnetic flux inside the disk and the normalized reference profile are presented in Figure 2.

## 3. Iterative Learning Control

Because of the sequential regime of the process of gathering the measurement data as a basic control scheme for the magnetic brake, a nonlinear iterative learning was employed. The choice of the ILC is justified not only by the repetitive character of the process, but is also dictated by its robustness to model inaccuracies and repetitive disturbances [9,21] via proper compensation with the use of the measurement data. This makes it especially useful, especially in combination with neural networks providing proven convergence for the considered class of systems [15].

Taking into account the fundamental property of the magnetic brake, namely the strictly energy dissipative character of the system (one cannot force the overshoot upwards), and in order to keep the complexity of the controller at a relatively low level, open-loop ILC was chosen so as to achieve the assumed control performance. Even though it is clear that the feedback control may improve the average performance, this may come at the cost of higher sensitivity to online disturbances.

**Figure 3:**
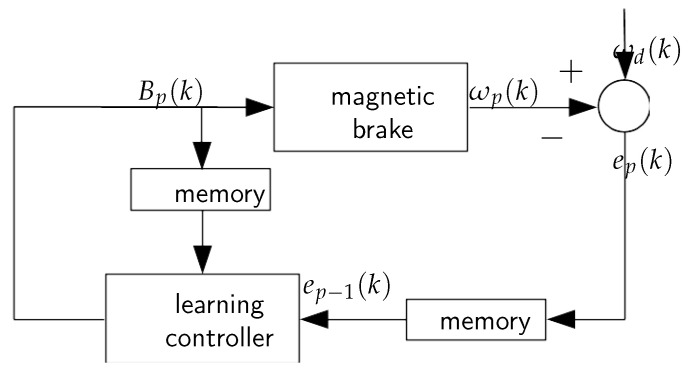
Iterative learning control scheme.

Because of the specificity of the controller learning process and neural modeling, it can be useful to further discuss the discrete-time domain. Thus, the general idea of the applied nonlinear ILC is represented by the scheme depicted in Figure 3. The learning controller has the following form:

(3) $$\begin{array}{c} B_{p}\left(k\right)=f\left(B_{p-1}\left(k\right),e_{p-1}\left(k\right)\right). \end{array}$$

where f(·,·) is a nonlinear function representing the dynamics of the controller, *p* stands for the iteration (trial) number, *k* is a time instant, $e_{p}\left(k\right)=\omega_{d}\left(k\right)-\omega_{p}\left(k\right)$ is the tracking error and $\omega_{d}\left(k\right)$ is the reference signal. The system (3) constitutes the so-called P-type first-order learning controller [9]. Various choices exists for effective realization of f(·,·). One of the possible solutions is to apply a feedforward neural network. The essential element of the learning algorithm is the necessity of estimating the output sensitivities of the magnetic brake with respect to the control signal. This is achieved using a model of the system as reported in previous works by the current authors [15,22,23]. The important feature of such a solution is the possibility to adapt its structure to the changing operation conditions of the control system. Due to the availability of representative measurement data, such an approach seems to be especially attractive here. As the details of the training procedure for the controller are beyond the scope of this paper, the interested reader can be referred to [15,22,23]. Although the physics of magnetic brakes can be discussed in terms of distributed parameter systems, such a class of systems can be accurately approximated with non-parametric models, such as a nonlinear state-space innovation form model (NSSIF):

(4) $$\begin{array}{cc} x_{p}\left(k+1\right) & =g(x_{p}\left(k\right),B_{p}\left(k\right),\epsilon_{p}\left(k\right)),\\ {\hat{\omega }}_{p}\left(k\right) & =Cx_{p}\left(k\right), \end{array}$$

where $x_{p}\left(k\right)$, $B_{p}\left(k\right)$ and ${\hat{\omega }}_{p}\left(k\right)$ are the state-space, input, and predicted output vectors, respectively, $\epsilon_{p}\left(k\right)=\omega_{p}\left(k\right)-{\hat{\omega }}_{p}$ is the prediction error and C is the output (observation) matrix.

**Figure 4:**
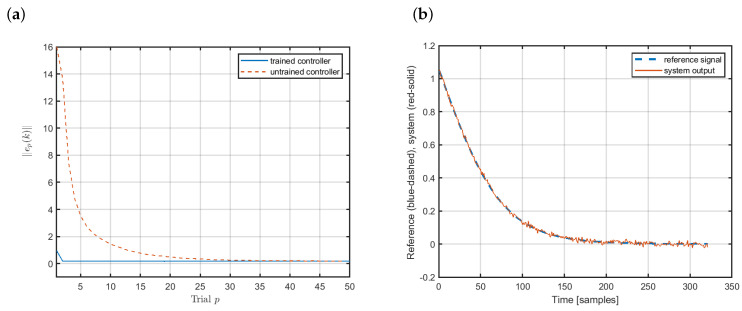
Control of the magnetic brake: (**a**) convergence of the tracking error norm and (**b**) tracking of the reference.

Figure 4 shows the control results relating to the tracking of the reference presented in Figure 2. Figure 4a illustrates the convergence of the tracking error norm for two cases: the untrained learning controller (red dashed line) and the preliminary trained controller (blue solid line). If we start with the randomly selected initial controller weights, the learning algorithm needs some time to adapt the parameters in order to follow the reference closely. Such an experiment is performed at the very beginning. After that we can use the preliminary trained neural controller, which is able to adapt to the current working conditions of the plant very quickly (blue-solid line). Figure 4b presents the quality of tracking of the reference, where the output of the the plant is marked with the red solid line while the reference is marked with the blue dashed line. Evidently, the applied ILC functions well and the plant output follows the reference closely.

The model (4) is in fact the state observer and works satisfactorily. However, in this paper we consider a fault-tolerant control design that relies on fault diagnosis. For the purposes of model-based fault diagnosis, a model of the system should be independent of the measured process variables. Unfortunately, the model (4) is dependent on the output of the system and thus cannot be used for fault detection and there a need for developing another model. This matter is taken up in the next section.

## 4. Model Design

As the NSSIF model cannot be used for fault diagnosis, our first attempt was aimed at the application of a state-space neural network represented by (4) assuming that the prediction error $\epsilon_{p}\left(k\right)=0$. However, due to the complex characteristics of the magnetic brake portrayed in Section 2, just one state-space model is insufficient to accurately follow the dynamics of the system at different operating points.

Therefore, in order to overcome this impediment, a reasonable idea is to express the dynamics of the system as a mixture of multiple models, where each of them describes the system around an operating point. The idea is reminiscent of the so-called ensemble averaging from machine learning theory and is closely related to the gain scheduling approach to nonlinear control. Although the NSSIF is known to possess the universal approximation property [15], the proper generalization of the system response is often achieved at the cost of the bias. The mixture of models, if properly adopted, can reduce the distribution of the model output still keeping a bias on the low level. As a result, we are able to determine a good trade-off between uncertainty modeling and robustness with respect to disturbances. However, to achieve this balance we have to properly combine the components of the approach, which will be discussed in the following sections.

First, if not leading to ambiguity, the trial index *p* is omitted for the clarity of the presentation. Then the overall model is represented as follows:

(5) $$\hat{\omega }\left(k\right)=\sum_{i=1}^{n}{\hat{\omega }}_{i}\left(k\right)\mu_{i}\left(\rho \right)$$

with

(6) $$\sum_{i=1}^{n}\mu_{i}\left(\rho \right)=1,$$

where $\mu_{i}$ are scaling functions, ${\hat{\omega }}_{i}\left(k\right)$ represents estimates of the system output at the *i*-th operating point, *n* is the number of operating points considered and ρ stands for the parameter embodying dependence of the combination of local models on the operating point that can be represented by input, state, etc. The formulation (5) is known as a nonlinear gain scheduling [24]. It can also be considered as an ensemble of models used for regression [25]. The functions $\mu_{i}$ can be perceived as a membership functions and then (5) looks similar to the Takagi–Sugeno representation. The crucial parts of the modeling are: (i) a proper designing of local models and (ii) proper choice of the membership functions. The first problem can be readily solved using the state-space neural network of the form:

(7) $$\begin{array}{cc} x_{i}\left(k+1\right) & =h_{i}\left(x_{i}\left(k\right),B\left(k\right)\right),\\ {\hat{\omega }}_{i}\left(k\right) & =C_{i}x_{i}\left(k\right), \end{array}$$

where the function $h_{i}\left(\cdot ,\cdot \right)$ describing the *i*-th neural model is represented by:

(8) $$x_{i}\left(k+1\right)=W_{i_{2}}\sigma_{h}\left(W_{i_{1}}^{x}x_{i}\left(k\right)+W_{i_{1}}^{b}B\left(k\right)\right)$$

where $\sigma_{h}\left(\cdot \right)$ is the nonlinear activation function of the hidden layer, $W_{i_{1}}$, and $W_{i_{2}}^{x}$ and $W_{i_{2}}^{b}$ are the *i*-th neural model weight matrices subject to training. Each neural network (7) is trained using data recorded during control of the magnetic brake at the specific operating point.

**Figure 5:**
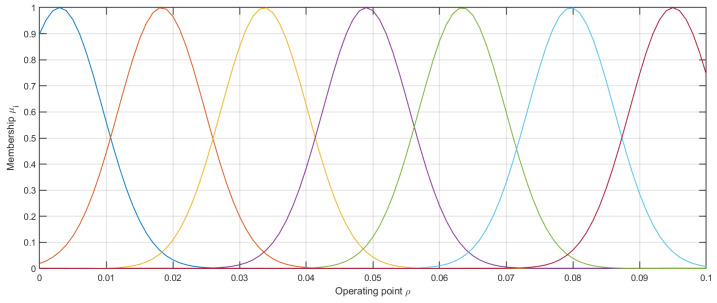
Distribution of the membership functions.

The latter problem can be effectively solved by means of Gaussian membership functions properly distributed on the domain ρ. In this paper, ρ is selected to be the operating point depending on the initial control value $B_{0}$. This choice is motivated by the fact that the behavior of the magnetic brake strictly depends on the initial conditions. One of them is an initial control value. Then:

(9) $$\mu_{i}\left(B_{0}\right)=e^{-\frac{\left(\right|B_{0}{\left|-B_{i}\right)}^{2}}{2\sigma^{2}}}$$

where $B_{i}$ is the *i*-th operating point and σ is the spread of the Gaussian function. In (9) the absolute value of $u_{0}$ is used because for the magnetic brake the total torque defined by (2) is invariant to the exchange of magnetic poles; thus we can restrict the considerations to nonnegative values of the magnetic flux only. Consequently, it was possible to reduce the number of operating points and then the number of local models required to develop the overall model (5). In the present work, it is assumed that each membership function has the same spread and they are uniformly distributed, cf. Figure 5. In this way, the condition (6) is always satisfied.

Throughout the empirical analysis of the magnetic brake system the effective range of the magnetic field varied from 0.003 to 0.095. After performing a series of preliminary experiments we decided to set the number of operating points to seven. The distribution of Gaussian membership functions is depicted in Figure 5.

In order to satisfy condition (6), the spread of the functions was set to σ=0.006512. The set of operating points was {0.003,0.0183,0.0337,0.049,0.0634,0.0797,0.095}. Data for model training were recorded during the normal work of the magnetic brake control system. For each operating point, one model represented by (7) was designed. The number of hidden neurons *v* as well as the order of the network $n_{x}$ were selected experimentally through trial and error. The final configuration of the models is presented in Table 1. A very important observation is that each local model has a relatively simple structure including a lower number of hidden neurons (only five) and model order (the second or third).

**Figure 6:**
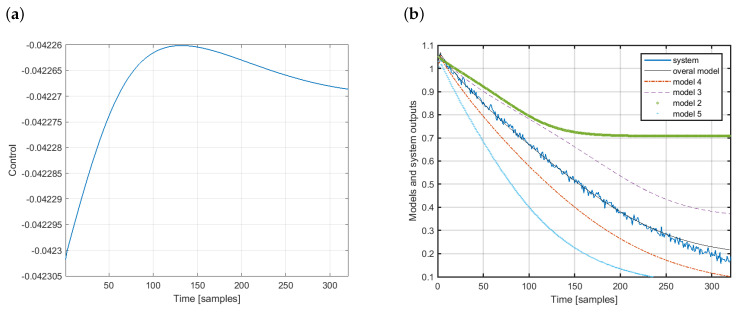
Modeling example: (**a**) Control signal and (**b**) output of models.

For illustration, let us analyze the modeling results presented in Figure 6.

In the case considered, the initial control was ρ=−0.0423. Then, four membership functions were activated with the following membership values: $\mu_{2}=0.0011$, $\mu_{3}=0.4179$, $\mu_{4}=0.5892$ and $\mu_{5}=0.0053$. This means that the estimated system output was calculated using four models. The third and fourth models had the most significant impact. The first, sixth and seventh models were not used here. Figure 6a shows the control signal. In turn, Figure 6b presents the output of the system (solid blue line) and the overall estimated output (solid black line) along with the outputs of all local models used to develop the overall model. Clearly, the proposed modeling design works pretty well. The sum of squared errors between the output of the system and its estimation is 0.0984. The model was tested on 100 sequences with different initial control conditions and the mean value of the sum of squared errors was 0.1681.

## 5. Sensor Fault-Tolerant Control

### 5.1. Fault Detection and Accommodation

Sensor fault detection is carried out on the ground of a residual signal. The residual is derived as a difference between the measurably available output of the system ω(k) and the output of the overall system model $\hat{\omega }\left(k\right)$:

(10) $$r\left(k\right)=\omega \left(k\right)-\hat{\omega }\left(k\right).$$

Using (10) the diagnostic signal in the form of the squared norm is proposed:

(11) $$d\left(r\right)=∥\tilde{r}{\left(k\right)∥}_{n}^{2},$$

where *n* is the length of samples in *r* and

(12) $$\tilde{r}\left(k\right)=\left(r\left(k\right)-\mu_{r}\right)/\sigma_{r}$$

with $\mu_{r}$ and $\sigma_{r}$ denoting the expected value and the standard deviation of the residual signal, respectively.

From a statistical point of view, this constitutes a sufficient statistic preserving information about the state of the system. In fact, assuming that the measurement noise is a zero-mean Gaussian white process and according to the form of membership functions given by (9), the diagnostic signal (11) is a random variable distributed according to the $\chi^{2}$ distribution with *n* degrees of freedom. In consequence, it is possible to use the diagnostic signal to construct a statistical parametric test to verify the null hypothesis about the excessive discrepancy of the norm from its nominal value. Thus, a decision about faults can be made. A very simple decision rule can be proposed, i.e., to compare the diagnostic signal d(r) with the suitably selected threshold *T*:

(13) $$s\left(r\right)=\left\{\begin{array}{cc} 0 & if\;d(r)\leq T,\\ 1 & otherwise. \end{array}\right.$$

In the ideal conditions (absence of measurement and modeling uncertainty) the threshold is zero in the fault-free case and different from zero in the case of a fault. However, due to the modeling uncertainty, disturbances and the measurement noise, it is required to assign a threshold larger than zero to avoid excessive numbers of false alarms. In order to determine the threshold, let us introduce a significance level α that corresponds to the fixed a priori probability of a false alarm. Then the threshold *T* can be determined as some critical value being approximately equal to the (1−α)·100% percentile from the cumulative $\chi^{2}$ distribution. When *n* is large, the random variable

(14) $$\tilde{d}\left(r\right)=\left(d\left(r\right)-n\right)/\sqrt{2n}$$

weakly converges to a standard normal distribution, so in practice, the determination of the threshold is even easier, as it is a solution to the equation

(15) $$\alpha =prob(\tilde{d}\left(r\right)>T),$$

which can be efficiently found from the tabulated values of the cumulative distribution function of N(0,1).

To make the threshold *T* applicable, it is required to determine the statistics $\mu_{r}$ and $\sigma_{r}$ of the residual signal. These can be derived by analysis of the residual signal derived in the normal (fault-free or healthy) operating conditions of the magnetic brake, as portrayed in Section 3. The important index assessing the fault diagnosis quality is the time of fault detection. In this paper we deal with a dynamic system that is used in a repetitive manner. Then, we propose to introduce a similar quantity to the time of fault detection called the trial of fault detection $p_{d}$. Clearly, the trial of fault detection is the trial number at which the diagnostic signal (11) permanently exceeds the predefined threshold (15).

After detecting a sensor fault, it is time for reconstructing the value measured by the faulty sensor. To this end, the overall model of a magnetic brake is used. Since the fault is detected, the output of this model replaces the value measured by the faulty sensor. However, the perfectly fitted model of the system does not exist. Then, it is necessary to estimate the modeling uncertainty somehow. Here, we decided to apply a very simple strategy that is easy to implement, in which the modeling uncertainty becomes constant from trail to trail during the fault occurrence and is estimated as:

(16) $$\Delta \omega \left(k\right)=\omega_{p_{d}}\left(k\right)-{\hat{\omega }}_{p_{d}}\left(k\right)$$

Finally, the reconstructed value of the system output can be derived as:

(17) $$\omega_{r}\left(k\right)=\hat{\omega }\left(k\right)+\Delta \omega \left(k\right)$$

### 5.2. Fault-Tolerant Control

In this paper, both the abrupt and incipient sensor faults are investigated. The abrupt fault is simply a sudden change of variables describing a sensor. When a fault occurs the value of a parameter jumps to a new constant value. In turn, the incipient fault gradually develops to a larger and larger value. The strength $f_{s}$ of the incipient fault can be modeled as follows:

(18) $$f_{s}\left(t\right)=\frac{t-t_{from}}{t_{end}-t_{from}}val$$

where $t_{from}$ is the fault start up time, $f_{end}$ is the fault end time and val is the fault strength at $t_{end}$. For the system working repetitively, this means that an incipient fault can develop through a number of subsequent trials. The faults can also be split into multiplicative and additive ones. The multiplicative fault is simulated as:

(19) $$\omega_{m}\left(t\right)=\omega \left(t\right)\left(1+f_{s}\left(t\right)\right),$$

where ω(t) is the real value of the process variable, $\omega_{m}\left(t\right)$ is the measured value and $f_{s}$ is the fault intensity. The additive fault is simulated adding some bias to the angular velocity sensor as follows:

(20) $$\omega_{m}\left(t\right)=\omega \left(t\right)+f_{s}\left(t\right).$$

In the case study the following faulty scenarios were investigated:

- scenario $f_{1}$—abrupt fault, multiplicative type, fault intensity: $f_{s}\left(t\right)=0.05$;
- scenario $f_{2}$—abrupt fault, additive type, fault intensity: $f_{s}\left(t\right)=+0.05$;
- scenario $f_{3}$—abrupt fault, additive type, fault intensity: $f_{s}\left(t\right)=-0.05$;
- scenario $f_{4}$—incipient fault (18), additive type (20), final fault intensity: val=0.05;
- scenario $f_{5}$—incipient fault (18), multiplicative type (19), final fault intensity: val=0.05.

**Figure 7:**
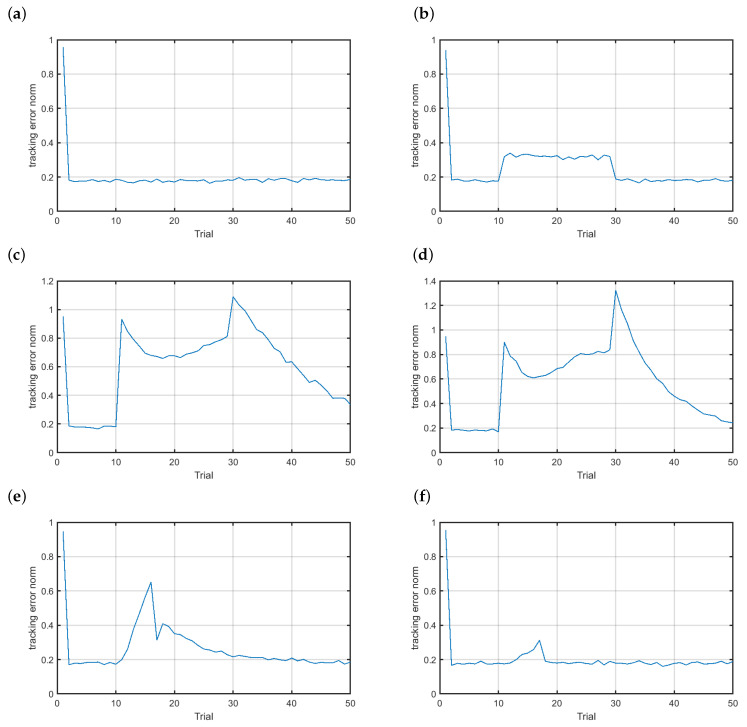
Control of the magnetic brake: (**a**) normal functioning, (**b**) scenario $f_{1}$, (**c**) scenario $f_{2}$, (**d**) scenario $f_{3}$, (**e**) scenario $f_{4}$ and (**f**) scenario $f_{5}$.

Each abrupt fault was simulated at the 11th trial and lasted for the following 20 trials. Figure 7 shows the behavior of the control system without fault tolerant abilities. In Figure 7a one can see the control performance of the healthy system. ILC drives the system to follow the reference closely with the tracking error norm below 0.2. Figure 7b displays the results in the case of scenario $f_{1}$. Clearly, the abrupt multiplicative fault with the intensity $f_{s}\left(t\right)=0.05$ significantly deteriorated the control performance. During the fault occurrence the tracking error norm increased to the level of more than 0.3. Even worse results were observed for the additive faults either with the positive or negative bias (Figure 7c,d). Clearly, any bias in the angular velocity sensor significantly brought down the control quality. The incipient faults were simulated at the 30th time instant of the 11th trial and lasted for the next 2000 time instants. This means that the incipient fault develops during the next seven trials. Figure 7e and Figure 7f show the control performance in case of the incipient scenarios $f_{4}$ and $f_{5}$, respectively. In general, additive faults had the stronger impact on the control performance relative to multiplicative ones. All presented scenarios clearly show that there is a need for applying a control system that renders it possible to accommodate faults and maintain the acceptable control performance in case of faults.

**Abrupt faults.** As mentioned earlier, each abrupt scenario was simulated at the 11th trial and lasted for 20 trials. Fault detection was carried out by means of the constant threshold (15). Then, the performance of the control system should first be evaluated during normal operation conditions. The control was carried out for the 100 trials, for each trial calculating the value of the diagnostic signal. After that the mean value as well as standard deviation were calculated to be:

$$\mu_{r}=-0.0226,\;\sigma_{r}=0.0344.$$

For the significance level α=0.05 the value of the threshold was T=1.644.

**Figure 8:**
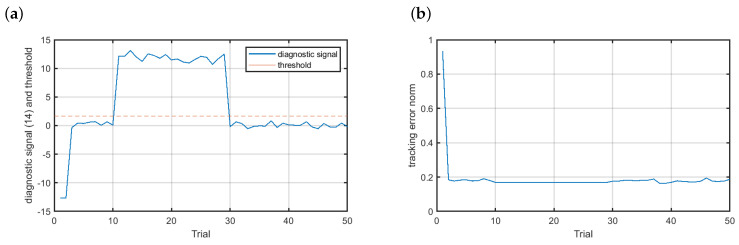
Fault-tolerant control—scenario $f_{1}$: (**a**) diagnostic signal (solid blue line) and the threshold (dashed red line), (**b**) control results.

In Figure 8a one can see the diagnostic signal (solid blue line) with the threshold marked with the dashed red line for the fault scenario $f_{1}$. Clearly, the fault is reliably detected with the trial detection index $p_{d}$ equal to 1. Due to the fact that ILC works offline, i.e., the control signal is derived based on data recorded during the previous trial, the fault accommodation can be immediately launched. The fault-tolerant control results are presented in Figure 8b. The fault was completely accommodated and the tracking error norm remained on the acceptable level. Additionally, the neural model had the property of data filtering. During training, the model weights are derived in such a way as to make it possible for the model to approximate any nonlinear mapping. That is the reason for the flat region observed in the tracking error norm course between the 11th and 30th trial.

**Figure 9:**
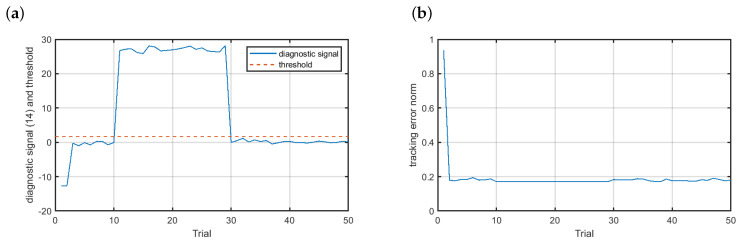
Fault-tolerant control—scenario $f_{2}$: (**a**) diagnostic signal (solid blue line) and the threshold (dashed red line), (**b**) control results.

Figure 9 shows the results for the faulty scenario $f_{2}$. The changes observed in the diagnostic signal (Figure 9a) were not as large as in the case of the multiplicative fault; however, this fault was also quickly detected with $p_{d}=1$, which renders possible the fast reconstruction of the signal measured by the faulty sensor while keeping high quality control of the magnetic brake.

**Figure 10:**
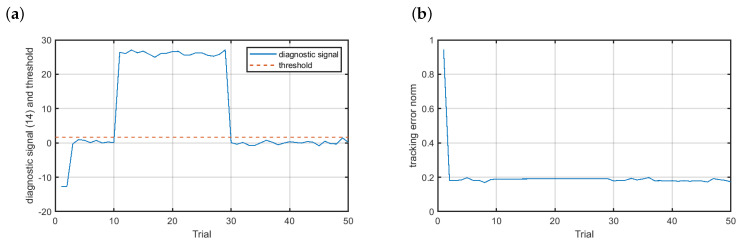
Fault-tolerant control—scenario $f_{3}$: (**a**) diagnostic signal (solid blue line) and the threshold (dashed red line), (**b**) control results.

The last abrupt scenario was the additive fault with negative fault intensity (Figure 10). Contrary to the previously analyzed scenarios, this time the fault effect was distinctly visible in the diagnostic signal course (see Figure 10a). As with the previous abrupt scenarios the fault accommodation was immediately launched. The fault was detected within one trial. The fault-tolerant control was satisfactory, as presented in Figure 10b.

**Incipient faults.** The incipient faults were introduced at the 11th trial. The fault startup time was set to the 30th time instant and the fault end time was the 2030th time instant. The full strength of incipient faults was achieved at the 17th trial. The threshold level used was the same as in case of abrupt faults.

**Figure 11:**
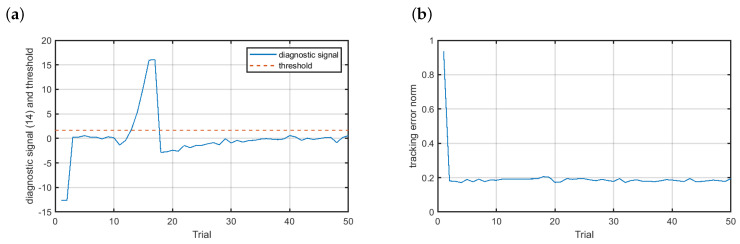
Fault-tolerant control—scenario $f_{4}$: (**a**) diagnostic signal (solid blue line), (**b**) control results.

Figure 11 presents the results achieved for the additive scenario $f_{4}$. As the fault developed slowly its detection took some time. In Figure 11a one can see that the diagnostic signal started to increase when the fault affected the velocity sensor. The diagnostic signal crossed the threshold at the 12th trial. This time fault accommodation launched one trial later than in the case of the abrupt faults. Nonetheless, the fault-tolerant control worked with a quality similar to the normal operating conditions. As a matter of fact, a slight increase of the tracking error norm was observable, but this change was not significant, contrary to the control scheme without FTC (compare with Figure 7e).

**Figure 12:**
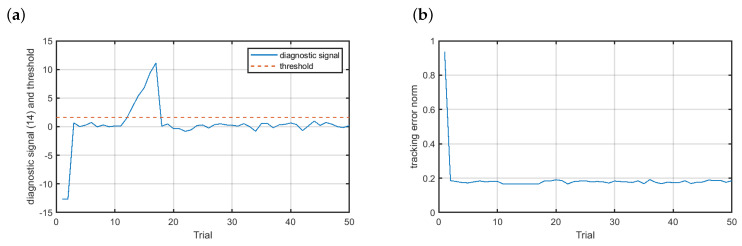
Fault-tolerant control—scenario $f_{5}$: (**a**) residual, (**b**) control results.

The last investigated scenario was the incipient multiplicative sensor fault. The obtained results are shown in Figure 12. It is clear that the evolution of the diagnostic signal was quite similar to that observed for the incipient additive fault $f_{4}$. The fault was accommodated at the 12th trial (Figure 12a). In means that the trail of fault detection was equal to 2. Despite the one trail delay of the reconstruction of the signal measured by the velocity sensor, the fault did not affect the quality of the control at all (see Figure 12b). This phenomenon can be easily explained by analyzing the evolution of the incipient multiplicative fault. From (18) we know that after one trial the fault intensity growed up to the value of 0.0073. For the multiplicative fault this means that the value measured by the velocity sensor increased by ≈0.007%. Such a fault intensity is not meaningful to the work of the control system.

The main objective of the FTC system is to maintain the current performance of the system as close as possible to the desirable one in the presence of faults. Then, the main performance index pointing out the quality of the proposed FTC system is the value of the tracking error. All reported experimental results clearly show that sensor faults were detected fast enough to assure high quality reference tracking. As shown in Figure 8b, Figure 9b, Figure 10b, Figure 11b and Figure 12b, the tracking error norm in case of a fault was kept on the level observed for the nominal operation conditions (see Figure 7a).

**Fault size influence.** To verify the performance as well as the sensitivity of the proposed FTC scheme the influence of the fault size on the control system performance was investigated. Key results are shown in Table 2. The abrupt/additive fault with a size smaller than 0.01 stayed undetected. For the abrupt/multiplicative fault slight problems were observed for the fault size val=0.01. In this case it was observed that for several trials the value of the diagnostic signal was below the threshold. However, the fault was marked as detected because during a predominant number of trials the diagnostic signal crossed the threshold. However, for a fault size equal to 0.008 serious problems were already observed as the diagnostic signal oscillated around the threshold and the fault was not detected anymore. For a fault size smaller than 0.006, the diagnostic signal remained below the threshold, indicating that the fault was undetected. In the case of an incipient fault, the smaller was the fault size, the longer was the fault detection time, which is expected behavior. For a fault size smaller than 0.007, the fault was not detectable. Generally speaking, the portrayed results clearly show that faults with an intensity of less than 0.01 were not detected by the proposed fault diagnosis procedure. Thus, the crucial question arises of what is the quality of the control when a fault occurred in a sensor and it was not detected. The simple verification is to compare the tracking error norm in the case of control with the presence of undetected fault with control at the nominal operating conditions. For example, for an incipient fault with a size equal to 0.007, the tracking error norm took the values from the interval [0.1666,0.1783]. Similar values were observed for the control at nominal operating conditions (see Figure 7a). Clearly, for the fault-free case, the tracking error norm converged to the level of the norm of disturbances acting on the magnetic brake. Then, a fault with an intensity of less than 0.01 influenced the system similarly to the external disturbance and had no adverse impact on the control system performance.

## 6. Concluding Remarks

The proposed method of sensor fault-tolerant control was found to be very efficient in the case of the class of nonlinear systems represented by the example of a magnetic brake. The advantage of the iterative control scheme is that the approach offers great flexibility to reduce the modeling and process uncertainty of nonlinear control design using the observational data available from previous trials. Despite its somewhat complex mathematical foundations, the resulting fault detection scheme is very easy to implement in practical conditions.

There is still space for further improvements, for example, to incorporate a feedback controller so as to increase the control performance with respect to online disturbances. More precisely, the offline ILC is not suitable for accurate compensation of random disturbances. In addition, the identification within a feedback loop is more difficult. As for a potential solution, the approach reported in [23] developed in the context of a similar class of nonlinear systems and feedback control can be extended to the online procedure.

Another potential future research direction that is very important in the context of neural modeling is the proper choice of representative observational data. A successful application of experimental design theory to solve this problem was developed in [22,26,27] but not in the context of FTC. The efficient adaptation of this approach establishes an open problem, which requires further investigation.

Yet another problem is an extension towards robust solutions with additional constraints imposed on the control design, e.g., related to the actuator faults or uncertainties in the material parameters. This can be solved within the framework of the proposed ILC scheme via exploration of dedicated structures of neural controllers.

## Figures and Tables

**Table 1:** Models configuration.

Model no.	$n_{x}$	*v*	$\sigma_{h}$
1	2	5	hyperbolic tangent
2	2	5	hyperbolic tangent
3	3	5	hyperbolic tangent
4	3	5	hyperbolic tangent
5	3	5	hyperbolic tangent
6	3	5	hyperbolic tangent
7	3	5	hyperbolic tangent

**Table 2:** Influence of the fault size on the fault diagnosis performance.

Scenario	Type	Size Val	$P_{d}$	Remarks
$f_{1}$	abrupt/multiplicative	0.01	1	At some trials the diagnostic signal was below the threshold
$f_{1}$	abrupt/multiplicative	0.008	undetected	Oscillations around threshold
$f_{1}$	abrupt/multiplicative	0.006	undetected	The diagnostic signal was permanently below the threshold
$f_{3}$	abrupt/additive	0.01	undetected	The diagnostic signal was permanently below the threshold
$f_{5}$	incipient/additive	0.02	5	—
$f_{5}$	incipient/additive	0.01	6	—
$f_{5}$	incipient/additive	0.007	undetected	The diagnostic signal was permanently below the threshold

## References

[1] Simeu E., Georges D. (1996). Modeling and control of an eddy current brake. Control. Eng. Pract..

[2] Zamani A. (2014). Design of a controller for rail eddy current brake system. IET Electr. Syst. Transp..

[3] Yang J., Yi F., Wang J. Model-based adaptive control of eddy current retarder. Proceedings of the 30th Chinese Control and Decision Conference.

[4] Lee K., Park K. (1999). Optimal robust control of a contactless brake system using an eddy current. Mechatronics.

[5] Anwar S. A torque based sliding model control of an eddy current braking system for automotive applications. Proceedings of the ASME International Mechanical Engineering Congress and Exposition.

[6] Xu Y.N., Deng W.W. (2015). Research of Multiple Sensors Adaptive Fault-Tolerant Control Based on T-S Fuzzy Model for EMB System. Int. J. Eng. Technol..

[7] Huang S., Zhou C., Yang L., Qinab Y., Huang X., Huab B. (2016). Fault-tolerant braking control with integerated EMBs and regenerative in-wheel motors. Reliab. Eng. Syst. Saf..

[8] Kim S., Huh K. (2016). Fault-tolerant braking control with integerated EMBs and regenerative in-wheel motors. Int. J. Automot. Technol..

[9] Bristow D.A., Tharayil M., Alleyne A.G. (2006). A survey of Iterative Learning Control: A learning-based method for high-performance tracking control. IEEE Control Syst. Mag..

[10] Chien C.J. (1998). A discrete iterative learning control for a class of nonlinear time-varying systems. IEEE Trans. Autom. Control.

[11] Blanke M., Kinnaert M., Lunze J., Staroswiecki M. (2016). Diagnosis and Fault-Tolerant Control.

[12] Noura H., Theilliol D., Ponsart J., Chamseddine A. (2003). Fault-Tolerant Control Systems: Design and Practical Applications.

[13] Ducard G.J.J. (2009). Fault-Tolerant Flight Control and Guidance Systems: Practical Methods for Small Unmanned Aerial Vehicles.

[14] Mahmoud M., Jiang J., Zhang Y. (2003). Active Fault Tolerant Control Systems: Stochastic Analysis and Synthesis.

[15] Patan K., Patan M. Neural-network-based high-order iterative learning control. Proceedings of the 2019 American Control Conference (ACC).

[16] Ponsart J.C., Theilliol D., Aubrun C. (2010). Virtual sensors design for active fault tolerant control system applied to a winding machine. Control Eng. Pract..

[17] Rotondo D., Nejjari F., Puig V. (2014). A virtual actuator and sensor approach for fault tolerant control of LPV systems. J. Process. Control..

[18] Tabbache B., Benbouzid M.E.H., Kheloui A., Bourgeot J.M. (2012). Virtual-sensor-based maximum-likelihood voting approach for fault-tolerant control of electric vehicle powertrains. IEEE Trans. Veh. Technol..

[19] Sami M., Patton R.J. Wind turbine sensor fault tolerant control via a multiple-model approach. Proceedings of the 2012 UKACC International Conference on Control, IEEE.

[20] Patan M. (2012). Optimal Sensor Networks Scheduling in Identification of Distributed Parameter Systems.

[21] Ahn H.S., Moore K.L., Chen Y. (2007). Iterative Learning Control. Robustness and Monotonic Convergence for Interval Systems.

[22] Patan K., Patan M. Design and convergence of iterative learning control based on neural networks. Proceedings of the European Control Conference, ECC 2018.

[23] Patan K., Patan M. (2020). Neural-network-based iterative learning control of nonlinear systems. ISA Trans..

[24] Leith D.J., Leithead W.E. (2000). Survey of gain-scheduling analysis and design. Int. J. Control.

[25] Sollich P., Krogh A. (1996). Learning with ensembles: How over-fitting can be useful. Adv. Neural Inf. Process. Syst..

[26] Patan K., Patan M., Kowalów D. Optimal sensor selection for model identification in iterative learning control of spatiotemporal systems. Proceedings of the 55th IEEE Conference on Decision and Control (CDC).

[27] Patan K., Patan M., Kowalów D. (2017). Neural networks in design of iterative learning control for nonlinear systems. IFAC PapersOnLine.

